# Extracts from medicinal plants inhibit cancer cell proliferation, induce apoptosis in ovary, lung and neuronal cancer cell lines

**DOI:** 10.1186/2049-3002-2-S1-P21

**Published:** 2014-05-28

**Authors:** Wafa Ghali, David Vaudry, Thierry Jouenne, Mohamed Nejib Marzouki

**Affiliations:** 1 Laboratory of Protein Engineering and Active Biomolecules, National Institute of Applied Sciences and Technology, University of Carthage, Centre Uurbain Nord, Tunis, Tunisia; 2 INSERM U982, Différenciation & Communication Neuronale & Neuroendocrine, Laboratory, URouen, IRIB, Mont-Saint-Aignan, France; 3 CNRS UMR 6270, Polymères, Biopolymères, Surfaces Laboratory, PISSARO proteomic facility, URouen, Mont-Saint-Aignan, France

## Background

For thousands of years natural products, especially plants and vegetables, have been used to fight against various diseases such as cancer, microbial infections and even neurodegenerative diseases. It has been shown that consumption of plants and vegetables have a direct influence on the proliferation, angiogenesis, metastasis of cancer cells.

## Materials and methods

Our work aimed to evaluate the antioxidant activity, protective properties and anti-proliferative capacity of *Lycium europeaum* and *Jatropha podagrica* extracts on the proliferation and evolution of A547 (ovarian cancer cell line), OVCAR-3 (human ovary adenocarcinoma cell line), A549 (Human lung adenocarcinoma cell line) and PC12 (rat adrenal medulla Pheochromocytoma cells). The cytotoxicity of the studied extracts was also evaluated on cerebellar granule cells. Cell viability was monitored using thiazolyl blue tetrazolium bromide (MTT) reduction assay. Induction of apoptosis in treated cells was measured through caspases 3/7 activity. Cytoprotective properties of the extracts were assessed by monitoring the effect of induced-oxidative stress damages on cellular macromolecules. Free radical scavenging capacity was investigated by different methods such as DPPH and reducing power.

## Results

Extracts were found to contain components that inhibit cell proliferation and display cytotoxic activity on cancer cells but not on normal cells. The cytotoxicity of the studied extracts was also evaluated on young cerebellar granule cells and found to be non-significant. The anti-proliferative activity appeared to be mediated by apoptotic mechanisms, as suggested by activation of caspases 3/7 following cell exposure to the extracts. *Lycium*, as well as *Jatropha* extracts, seemed to be able to initiate cellular pathways that led to apoptosis when added to tumoral cells but did not show a significant cytotoxicity in contact with normal cells. Results also showed that the extracts protected lipids, proteins and DNA against oxidative stress damages induced by H2O2 via scavenging ROS. The antioxidant capacity of the studied plant extracts was correlated with the anti-proliferative activity.

## Conclusion

The present data demonstrate that *Lycium europaeum* and *Jatropha podagrica* may inhibit the proliferation of cancer cells and induce apoptosis and could provide protection from oxidative stress diseases thanks to their high antioxidant molecules content.

